# ‘Just stuff yourself’: Identifying health‐promotion strategies from the perspectives of adolescent boys from disadvantaged neighbourhoods

**DOI:** 10.1111/hex.12913

**Published:** 2019-06-14

**Authors:** Eva Lems, Femke Hilverda, Jacqueline E. W. Broerse, Christine Dedding

**Affiliations:** ^1^ Athena Institute for Research on Innovation and Communication in Health and Life Sciences Vrije Universiteit Amsterdam The Netherlands; ^2^ Amsterdam UMC, Medical Humanities Vrije Universiteit Amsterdam Amsterdam The Netherlands

**Keywords:** adolescents, disadvantaged groups, gender, health promotion, participatory action research

## Abstract

**Context:**

The prevalence of overweight and obesity among adolescents has risen dramatically in the last decade, disproportionally affecting adolescents from disadvantaged neighbourhoods. Adolescent boys from disadvantaged neighbourhoods are hard to reach for health promotion.

**Objective:**

This study aims to understand perceptions of health and health‐promotion strategies among adolescent boys from disadvantaged neighbourhoods in order to identify opportunities for health promotion that are better tailored to their needs.

**Methods:**

A qualitative, participatory research approach was used. Sixty‐three adolescent boys (aged 12‐18) were recruited from disadvantaged neighbourhoods in Amsterdam, the Netherlands. Semi‐structured interviews, participant observations and co‐creation sessions were conducted. Data were analysed using ethnographic content analysis.

**Results:**

Boys associate the consumption of large portions of unhealthy foods, especially meat, with masculinity and autonomy. Buying junk food is an important part of their social lives. According to boys, current health promotion does not fit their needs. They stress that entertaining activities, humour and short‐term benefits of healthy choices must be central to health promotion. Some differing interests in health promotion appear between boys, but all boys plead for cheap, satisfying, tasty and healthy food options in their neighbourhoods.

**Conclusions:**

Adolescent boys from disadvantaged neighbourhoods do see opportunities for health promotion. There is an emerging acceptance of boys taking care of their body and health, but the social norm of unhealthy consumption dominates. For health promoters, it is vital to gear health messages to who the boys are and wish to be, especially in relation to their peers.

## 1 INTRODUCTION

The prevalence of overweight and obesity among adolescents poses a serious public health threat worldwide.^1^ Around a third of European adolescents are overweight. This is an enormous problem, since 80% of these adolescents are likely to remain overweight for the rest of their lives.^2^ Overweight during adolescence is associated with many physical and mental health problems.^3^ Adolescents tend to have unhealthier lifestyles (ie high consumption of fast food, sugary beverages and insufficient physical activity) than people from other age groups.^2^, ^4^ This means that it is essential to promote a healthy lifestyle during adolescence; however, effective (obesity) interventions for adolescents are scarce.^5^ Health promotion is particularly important for adolescents with a low socioeconomic position since overweight and obesity are more prevalent in this group.^6^

According to the World Health Organization,^2^ socioeconomic position (SEP) accounts for 27% of the risk of overweight European adolescents, including those in the Netherlands. In disadvantaged neighbourhoods (neighbourhoods with on average lower incomes, more unemployment, poverty and lower education level compared to other neighbourhoods) in Amsterdam, 29% of adolescents with a low SEP are overweight or obese, compared to 14% of adolescents with a higher SEP.^7^ Several studies have found that the daily diet of adolescents with a low SEP diverges further from dietary recommendations compared to wealthier adolescents.^8^, ^9^ Although adolescents with a low SEP are known to be most at risk of becoming overweight, there is a lack of effective interventions.^10^ It has been suggested that interventions might even increase the gap between adolescents with a low and high SEP, since these are more effective among the latter.^11^ A review of obesity‐prevention strategies among low SEP adolescents^10^ found no clear evidence for effective strategies.

Besides SEP, gender also affects the likelihood of overweight in adolescents. Girls seem to take advantage of health interventions more often than boys.^11^ Although there are strong indications that gender influences lifestyle and health,^12^, ^13^ research into adolescent (over)weight often overlooks gender differences.^12^, ^13^, ^14^, ^15^ Many studies focus on girls in relation to overweight and interventions to prevent this, as shown in a review by Spencer et al,^16^ but there is comparatively little research on adolescent boys.^17^, ^18^ Research shows gender differences in health‐related behaviour; for instance, girls tend to eat more healthfully than boys—more fruit and vegetables,^2^ less junk food^18^ and fewer sugary beverages.^19^ Girls may eat healthier and show more interest in healthy eating and dieting because they want to be slim and align with Western beauty ideals.^20^ According to Stok,^21^ this is why girls are often more open to joining healthy lifestyle interventions.

Many studies suggest that boys are more physically active than girls.^2^ In general, boys are more satisfied with their weight, which they tend to underestimate,^22^ but are just as dissatisfied as girls with their body overall.^23^ Rowlands and Gough^14^ argue that due to ‘perfect’ masculine bodies in (social) media, there is growing pressure for men to strive for a muscular body. This beauty ideal is complex, as the dominant social expectation is that boys should be muscular, but neither ‘fat’ nor ‘skinny’.^14^, ^16^

Taking into consideration that overweight and obesity in adolescents are influenced by both gender and social background, tailored interventions may be most suitable and effective. In order to develop a tailored health promotion, however, there is a need for insights into how these boys perceive their health and lifestyle, as well as what opportunities they see for health promotion. There are multiple arguments for a participatory research approach. First, it is the right of children and adolescents to participate in matters affecting their health and lives as stated in the Convention on the Rights of the Child of the United Nations. Second, participation increases feasibility and validity of research, and eventually the chance of designing successful interventions.^21^, ^22^, ^24^ In addition, participation in this type of research contributes to personal development and self‐efficacy among young people by prompting them to reflect on their lives and take shared action.^25^

The aim of this article was to understand the perceptions of health and health‐promotion strategies among adolescent boys from disadvantaged neighbourhoods in Amsterdam, the Netherlands, in order to identify opportunities for health promotion that is better tailored to their needs.

## 2 METHODS

The article presents a participatory study conducted between November 2016 and May 2017 with adolescent boys. This study is nested in a larger Participatory Action Research (PAR) project (2015‐2018) aiming to improve health promotion (policy) with/for adolescents (boys and girls) with a low SEP in Amsterdam (the Netherlands), commissioned by the municipality of Amsterdam (Amsterdam Healthy Weight Program [AHWP]).

The PAR approach is particularly useful in studying and involving hard‐to‐reach groups, such as adolescents with a low SEP and/or poor literacy, and emphasizes understanding and listening to the voices of groups that are seldom‐heard.^26^, ^27^ PAR involves data collection, reflection and taking action to achieve public improvements in close collaboration with the participants.^26^, ^27^ PAR is an iterative and flexible process, which makes it possible to make changes in order to adjust to the participants' needs and preferences.^27^, ^28^

To gain insights into the perceptions and ideas for health promotion of adolescent *boys* with a low SEP, we organized several participatory research activities. In total, 63 adolescent boys (aged 12‐18) from two disadvantaged neighbourhoods in Amsterdam joined. The following activities were performed:

1. Nine individual interviews and three duo interviews with boys (n = 15) to gain insight into their perceptions of health and their ideas for health promotion in order to advise health policymakers.
2. Participant observations (30 hours) at existing social and health‐promotion activities organized by youth welfare centres (cooking evenings) and a school (workshops about food during a lifestyle‐themed week), aiming to gain insight into boys' (n = 27) behaviours/attitudes during health‐promotion activities.
3. Two preparatory co‐creation sessions (n = 11) and six co‐creation sessions (n = 10) as part of a small pilot intervention (‘The Healthy Lifestyle Project’) at a youth welfare centre, designed with boys and local youth workers. Sports and cooking activities were combined in six co‐creation sessions over a 3‐month period of time, aiming to challenge boys to reflect on their own lifestyles and advise policymakers and other professionals about health promotion that fit their daily realities.

**Figure 1:**
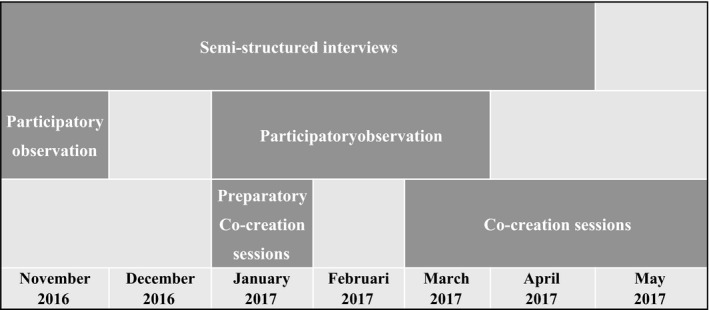
Timeline of the research activities

By using different methods, a comprehensive understanding and triangulation of data were ensured. The first participant observations were used to prepare for the interviews and preparatory co‐creation sessions. The later participant observations were used to deepen and validate interviews and vice versa. Both interviews and observations provided input for the (preparatory) co‐creation sessions. Figure 1 provides a timeline of the performed activities.

All research activities were performed by the first author (EL), who has experience in the field of public health and youth participation. The project was supervised by a senior researcher (CD) who is an expert in PAR/ethnography, especially with children and adolescents.

### 2.1 Recruitment

Participants were recruited from two secondary schools (practical training level or vocational education secondary school level) and three youth welfare organizations in two disadvantaged neighbourhoods in Amsterdam. These neighbourhoods were selected because they have many low‐income families and there is a high rate of overweight and obesity.^7^ Besides education level and living in a disadvantaged neighbourhood, age between 12 and 18 was a selection criterion. To ensure inclusiveness, avoid stigmatization^29^ and have a diverse group of participants, body size and current lifestyle were not selection criteria.

Generally, recruitment of hard‐to‐reach groups (including adolescents with a low SEP) is difficult; therefore, multiple recruitment strategies were used to maximize recruitment success^29^:

1. For the interviews, boys were recruited by a teacher at one school. In four school classes (all grades were represented), boys were asked whether they wanted to volunteer for an interview. At the other school, boys were recruited via the teacher of an after‐school sports programme for inactive students. Two boys were recruited by the researcher during participant observations at a youth welfare centre.
2. The locations and activities for the participant observations were found via the network of the municipality of Amsterdam. The researcher introduced herself during these activities and explained the research.
3. For the preparatory co‐creation sessions, boys were recruited by two youth workers and a teacher (known via the network of the municipality of Amsterdam). For the final co‐creation sessions, local youth workers and the researcher asked boys—face to face or via WhatsApp—to participate during activities at the youth welfare centre, as well as through leaflets and Facebook messages.

### 2.2 Participants

Most participants joined school at the practical training level or vocational education secondary school level and were of a non‐Western background. Most seemed to have a healthy weight based on the estimation of the researcher. Table 1 provides a description of the boys participating in interviews and co‐creation sessions. It was not possible to collect full background characteristics of boys consulted during observations.

**Table 1:** Description of boys participating in semi‐structured interviews and co‐creation sessions

	Semi‐structured interviews	Preparatory co‐creation sessions	Co‐creation sessions
Number of participants	15	11	10
Age (ranged 12‐18 y)	Mean = 14.1	Mean = 14.8	Mean = 13.9

	Frequency (percentage)	Frequency (percentage)	Frequency (percentage)
School level			
Practical training level	10 (67%)	4 (36.5%)	4 (40%)
Vocational education secondary school level	4 (27%)	‐	5 (50%
Intermediate vocational education	1 (7%)	3 (27%)	‐
Unknown	‐	4 (36.5%)	1 (10%)
Overweight^a^			
Yes	5 (33%)	3 (27%)	2 (20%)
No	10 (66%)	8 (73%)	8 (80%)

a Based on the estimation of the researcher.

### 2.3 Semi‐structured interviews

Semi‐structured interviews took, on average, around 45 minutes. Topics and sample questions are shown in Table 2. Boys from school were allowed to miss part of their lessons and received a small gift (eg deodorant). Boys from welfare organizations received a voucher for 7.50 Euros as compensation for their time. Interviews were held individually (n = 9) or with a friend (n = 6). The interview design was adjusted to the boys' age and low health literacy. Visual tools and creative exercises (eg responding to provocative statements by means of red/green‐coloured cards and placing pictures in order of importance) were used to make it easier for the boys to express themselves. There were always opportunities during the interviews for boys to introduce topics (see Table 3 for themes brought up by the participants). Interviews were recorded. Two boys did not wish to be recorded, so extensive notes were made.

**Table 2:** Research topics and example questions during research activities

Topic	Example questions
Perception of health	What are the most important things in your life? Where do you position ‘health’ between those things? Why? What does ‘being healthy’ mean for you? What pops up in your mind? ‘Only girls care about healthy lifestyle’ (agree/do not agree, why?)
Lifestyle	What are your healthy habits? What are your ‘lifestyle killers’? People differ from each other, boys might differ in lifestyle. What ‘types’ of boys do you think of? What type are you? Explain
Barriers	Why do many boys eat unhealthily or do not engage in any sport? What makes it difficult to make healthy choices? Why?
Motivators	Why do you/would you make healthy choices? What would motivate your friends? What would you tell a friend who eats junk food, does not do any sport?
Opportunities for health promotion	‘Teenagers just have an unhealthy lifestyle. There is nothing you can do about it’ (agree/do not agree, why?) What should school/municipality/parents/youth (health) workers/health educators do to motivate boys to make healthy choices?

**Table 3:** Main themes brought up by participants

Themes regarding (healthy) lifestyle	Themes regarding health promotion
Differences between boys	Own responsibility
Short‐ versus long‐term benefits	Role models
Peer influence/social status	Fun
Price of food	Practical use
Taste	Attractiveness of healthy food
Feeling satisfied	Having a say
Culture (cultural norms)	Fitting to lifeworld
Influence of school and neighbourhood	
Home situation/parents	
Physical attractiveness	
Sports performance/muscles and nutrition	
Feeling good	

### 2.4 Participant observations

Participant observations took place during a lifestyle week at a practical training school and five cooking evenings at two youth welfare centres. The researcher (EL) joined health‐promotion activities and observed^30^ how boys behaved during the activities and how they talked about it. The researcher also talked with boys (n = 27) about their perceptions of health (promotion) during and after activities. To reduce researcher bias, extensive field notes were made and discussed afterwards with youth workers who led the activities.

### 2.5 Co‐creation sessions

First, two preparatory co‐creation sessions (on average about 1.5 hours) were held to identify health‐promotion strategies. In a session at a practical training school, participants (n = 4) were challenged to create leaflets aimed at motivating peers to participate in after‐school sports activities. During a session at a youth centre, participants (n = 7) were asked to advise the municipality of Amsterdam about measures to improve the lifestyles of adolescents. Girls joined in these preparatory sessions, but only issues related to boys were used in the preparation of the final co‐creation session.

Six final co‐creation sessions were held during the ‘Healthy Lifestyle project’. Sessions were led by researcher EL together with a youth worker. Although the researcher directed the project, boys were involved as partners, deciding on activities (eg which sports and what to cook) and themes to discuss (eg protein shakes). In total, ten boys joined; some (n = 2) joined all of these sessions, others (n = 8) once or twice. On average, four boys per session joined.

Scripts for the sessions were based on the research questions and input of boys during earlier sessions (see Tables 2 and 3). During every session, advice and ideas were collected by the researcher. The final session was used to present and discuss the main findings with the boys. Since the boys were not eager to present their ideas themselves to policymakers, the researcher did. Owing to their dynamic structure, it was not useful to record during the (preparatory) co‐creation sessions. Extensive notes were made and discussed with the attending youth worker to check for discrepancies and omissions.

### 2.6 Data analysis

Interviews were transcribed verbatim. All transcripts, notes and created content from the co‐creation sessions were imported into MAXQDA2007 qualitative data‐analysis software. Ethnographic content analysis was used to identify and understand the relevance, significance and meaning of the data.^31^ Analyses were conducted during and after data collection. The use of a log, repeated review of the data, and seeking feedback from researcher colleagues and involved youth workers on content as well as possible projections and blind spots of the researcher enlarged reflexivity and reduced researcher bias.^32^

During the study, data were coded by researchers EL and CD. First, transcripts and field notes were read to become familiar with the data and code for recurring themes (inductive). Then, codes were discussed by the research team, refined and organized into overarching (sub)themes by looking for emergent patterns and themes. The codebook was revised and refined during the project until data saturation was reached (no new themes emerged from the analysis). Once data saturation was reached, we stopped recruiting participants. Midway through the analyses, the main outcomes were checked with participants (‘Tell me what I may be missing’) to deepen our understanding and enhance the credibility and trustworthiness of our findings (Berger, 2015).^32^ Table 3 shows the final main themes brought up by the participants.

## 3 RESULTS

As expected, it was difficult to recruit adolescent boys from disadvantaged neighbourhoods. There was very little interest in the topic of a healthy lifestyle, and boys were reserved/suspicious about joining the research activities. The researcher took time to connect with the boys, build up trust and show respect for who they are, regardless of their lifestyle choices, by patiently hanging around with them. Despite the lack of initial interest, a total of 63 boys were willing to share their perspectives, barriers, motives and advice on healthy living. Names mentioned in this result section are pseudonyms to protect the anonymity of the participants. Quotes are translated from Dutch to English by the authors.

### 3.1 Perspective on healthy living

For most boys, living healthily means taking care of your body (eat enough, drink water, no junk food, no drugs or alcohol) and looking good (not being overweight, no pimples and dark rings). Boys also consider mental and social aspects important: avoiding stress, spending time with friends and having enough money are seen as part of a healthy lifestyle. Although most boys find their health important, they have many unhealthy habits. In particular, their eating habits do not meet dietary recommendations (eg they eat junk food and drink energy and sugary drinks on a daily basis). Many boys explained that for boys, it is normal to spend 5‐10 Euros on food/drinks in supermarkets, fast‐food outlets or the school canteen on a daily basis. Boys often said that as long as they do not experience direct negative consequences of their unhealthy lifestyle, they will not change their unhealthy habits such as eating junk food. Though at first sight the boys seemed indifferent to healthy living, all of them rejected the statement ‘Only girls care about healthy lifestyle’. Boys care too, but there are differences between boys in their interest in a healthy lifestyle. Four ‘types’ of boys were distinguished: ‘Soccer boys’, ‘Fitness boys’, ‘Gamers’ and ‘Hang‐around boys’ as shown in Table 4. These profiles mainly differ in their interest in sports and the importance of appearance.

**Table 4:** Differences between boys in lifestyle

	Exemplary quotes	Typical lifestyle	Potential healthy lifestyle triggers
Soccer boys	‘They should start a [soccer] tournament’ (Sven, 12 years) ‘They only think: sports, sports sports’ (Orlando, 16 years)	Do lot of physical exercise, enjoy sport, especially playing football Usually eat dinner at home. Consume energy drinks and many snacks and sweets	(Famous) sport players as role model Health messages about what food improves sports performance Health promotion combined with sport tournaments/challenges
Fitness boys	‘You go to the gym for your own goals, mine is getting more muscles’ (Amir, 18 years) ‘“Shakies” go to the gym often and drink [protein]shakes’ (Baz, 14 years)	Go to gym often, enjoy other sports too Eat a lot, often meat and junk food, and protein shakes Looks are important	Sporty/masculine role models Health messages about what food you should eat to have an attractive (muscular) body Present healthy protein‐rich food options
Gamers	‘I would rather sit in my room to eat and play music than to go outside for sports’ (Ayoup, 15 years) ‘They find physical education useless’ (Vincent, 14 years)	Do not like physical exercise/sports Consume a lot of sugary beverages and junk food, mostly at home Are more often overweight than other types of boys	Role models with same interests (like gaming or arts) and not with a perfect body Fun, non‐competitive physical exercise activities, not like sport Use gaming/arts to get attention
Hang‐around boys	‘I don't eat leaves [spinach]…Men are hunters’ (Hamed, 16 years) ‘They say: “Check my nice clothes and Lois Vuitton bag”. That really has to do with showing off’ (Mike, 16 years) ‘They want to have a well shaped body, but they don't want to do any effort’ (Orlando, 16 years)	Like to chill and hang around on the street Buy a lot of junk food on the street Very little interest in health messages Looks are important	(Local) masculine male role models Do not mention ‘health’ in health promotion

### 3.2 Barriers

Boys experienced several barriers and motives in relation to living healthily, which are summarized in Tables 5 and 6. Some of these barriers and motives hold true for all boys, but others may differ, as demonstrated in the quotes. The most important barrier for most boys to live healthily was their perception that *healthy food harms social identity*. Eating healthy food can damage their image while eating large portions of meat and junk food reinforces social relations and boosts the desired characteristics of masculinity and independence. However, some boys explained they do make deliberately healthy choices. For example, Carl (aged 17, healthy weight) explained that when he eats a lot of junk food in one week, the following week he eats dinner at home and drinks more water, but that he will never tell his friends, because ‘that would be very awkward’. Among themselves, boys do not seem to talk about eating healthily.

**Table 5:** Main barriers to a healthy lifestyle

Barriers	Quotes
Eating healthily harms social identity	‘Only first‐graders eat sandwiches from home’ (Carl, 17 years, healthy weight)
Healthy food is too expensive	‘For a Euro I can buy four donuts, for a Euro I cannot even buy a small cup of healthy yogurt drink, because that is already 1.25 Euros’ (Ayoup, 15 years, obese)
Healthy food is not filling enough	‘Boys just want to eat a lot of tasty food. Eating healthily has nothing to do with price. Boys just don't not think it is necessary to eat healthily. They just think: ‘I'm hungry, I need to stuff myself’’ (Orlando, 16 years, healthy weight)
Unhealthy physical environment	‘They say it [school canteen] is a ‘healthy canteen’, but they still sell pizza and other unhealthy stuff’ (Mike, 16 years, overweight)
Unsupportive social environment:	‘I like it [fruit] but I don't buy it often. When I'm at home I don't feel like going to the supermarket anymore to buy it. EL: Is there fruit at home usually? Sometimes my dad buys it but no, not always’ (Carl, 17 years, healthy weight)

**Table 6:** Main motives to lead a healthy lifestyle

Motives	Quotes
Being attractive: Not being fat	‘If you want to lose weight, you don't eat unhealthy stuff…you don't want to get fat’ (Ravin, 13 years, healthy weight) ‘If you eat unhealthily, you get fat and then your life is over’ (Robin, 12 years, healthy weight)
Being attractive: A skin without pimples	‘Since I found I get pimples from greasy food, I only eat Kapsalon^a^ at weekends’ (Mo, 17 years, healthy weight)
Being attractive: Muscular body	‘I go to the gym because I want to get bigger…because of training I went from 60 to 80 kilo…EL: Why do boys go to the gym? You want to look different [more muscles or lose weight] then you feel better’ (Amir, 18 years, healthy weight)
Improved sport performance	‘Since I lost a lot of weight, I notice I can run faster and I'm a better striker. First I always had to be goalkeeper’ (George, 12 years, healthy weight)
Physical well‐being	You do sports, to be able to lift heavy things. And to feel better, that is because of hormones released during sports’ (Vincent, 14 years, healthy weight) ‘Water is better if you are thirsty than soda. And soda makes you fart a lot’ (David, 12, healthy weight)
Mental well‐being, having energy/having fun	‘You don't want to wake up and think ‘I have no energy’’ (Orlando, 16 years, overweight) ‘I play football with my friends just because I really like it’ (Amir, 18 years, healthy weight) ‘I only like sports if it is like playing and when I have fun with others’ (Ayoup, 15 years, obese)

a Dutch food item with chips, shawarma meat and cheese

Second, the majority of the boys emphasized that healthy food is *too expensive* and *is not satisfying* compared to junk food. Boys often said they need to eat a lot, especially meat, because they think that their body needs it. After eating healthy foods such as fruit, vegetables or a wholegrain toast, they still feel hungry.

The third barrier for all boys was their *social and physical environment*. There are many accessible, cheap, fast‐food outlets in the neighbourhood. Boys argued that none of them will stop eating junk food as long as fast‐food outlets are so prevalent. Some boys say that they lack places to play safely and play football. Boys with a Surinamese background stress that it is hard to make healthy choices because their food culture contains many fried dishes and their beauty ideal is voluptuous; people say you look sick if you are slender.

Finally, many boys highlighted the important role of their parents, who they believed should set a proper example. Although some boys said that their mothers cook vegetables, others said that their parents do not eat healthily or that there is no fruit at home. In addition, some boys said that if they do not like what is being served at home, they get money from their parents to buy something else—which could explain the large expenditure of many boys on junk food.

### 3.3 Motives

Most motives related to wanting to *be attractive* to girls. This means not being fat, being muscular and having good skin without pimples. Boys with visibly (serious) overweight suffered from bullying, which was their main motive for losing weight. George, aged 14, who recently lost a lot of weight, said that he just wanted to be ‘normally skinny’ before going to high school. Other important motives were the *direct effects on mental and physical well‐being* and *improved sports performance*.

### 3.4 Opportunities boys see for health promotion

Most boys primarily hold themselves responsible for their lifestyle choices, as one of the boys explained during a cooking workshop, ‘Whether you become fat or not, that depends on yourself’. Nevertheless, boys saw opportunities to (improve) health promotion for adolescent boys. First, they argued for cheaper, tastier and healthier food in their schools and neighbourhood. This is an important opportunity for health promotion.

Second, some boys explained that existing health education needs to be improved because it does not correspond with their lives, interests and cultural background. ‘Why do they [health educators] bring cucumber with hummus every time? We do not eat that stuff here,’ wondered one of the boys. In addition, many boys cannot identify with health educators: ‘I hate it when skinny females just say what I have to eat’ (Ayoup). Some boys suggested that it would be better if local role models or peers informed them. Vincent: ‘Then you think: that boy is cool and he managed (to lose weight), a small light will go on.’

Apart from who provides health information, boys had a lot of advice to give on the content of health education. Messages should not be like teaching, but fun, humorous, short and positive. The content must be relevant, focusing on direct positive effects. Some boys wanted to know what you should eat when you do weight training. Overweight boys, in particular, wanted to know how to lose weight. Some boys also mentioned a need for information about the deceitful practices of the food industry (for instance, saying that orange juice is healthy even though it is full of sugar).

Third, all boys emphasized that developing a healthy lifestyle app for adolescents would be a waste of money. Nobody would use it. Ayoup tried a weight‐management app: ‘I used it for two days, but then I stopped, it was too boring.’ Making an app fun like a game is not recommended, because then the boys would only play for fun and would not learn anything from it. In contrast, many boys thought short (at most 2.5 minutes) vlogs via Facebook and YouTube have potential for health promotion, because this fits their daily activities and does not require much effort from them. These vlogs should be funny and recognizable. The vloggers do not have to be famous—local role models can work just as well or better than celebrities.

Fourth, the boys often said that it is important to not push healthy choices and use fun activities. Not all boys like sport‐related activities; however, all of them enjoyed cooking activities (which were combined with food education and discussion/reflection). Mike says: ‘The cooking part is most fun; you learn and you eat together.’ Many boys thought that free cooking classes are a suitable way to convey food education. Most essential for boys is that they have a say in the activity, for instance, that they can choose recipes themselves and play their own music. Some boys did not want to join a vegetarian‐burger cooking workshop, so there should be an opportunity to cook with meat.

## 4 DISCUSSION

This study is one of the first to consider the perspectives of adolescent boys from disadvantaged neighbourhoods on health promotion and to explicitly ask them for their input on how to provide it. At first glance, boys show little interest in the topic of a healthy lifestyle and are very sceptical about health promotion. Boys experience many barriers to living healthily, mostly concerning healthy eating habits (fruit, vegetables, no junk food and energy/sugary drinks). Barriers in relation to other health‐related issues, such as physical exercise, stress and sleep, were mentioned less often. Healthy eating is especially difficult because it can affect boys' social status, and unhealthy food is more satisfying and cheaper than healthy food. The challenge is to translate these needs into health promotion, for example by stressing satisfying yet healthy food options and social marketing campaigns by role models.

This alone is probably not enough to change behaviour, however. Boys point to their social and physical environment: their neighbourhoods have a lot of unhealthy food options and local cultures discourage healthy choices. These findings are in line with ecological frameworks for health promotion, which underline the major cultural and environmental influences on health,^33^ for example, showing the significant influence of socioeconomic position on health‐related behaviour. The boys in our study, who live in relatively poor neighbourhoods, mention poverty only indirectly by emphasizing that healthy food is too expensive and activities should be free of charge. Price is important for the boys in making (food) choices. This contradicts the finding that boys spend a considerable amount of money on food every day, instead of taking a packed lunch from home or having dinner at home. Compliance with the social norms, which include eating in fast‐food outlets and the lack of healthy food at home, might explain this contradiction.

There seems to be another contradiction. Boys not only blame their environment for eating unhealthily but also hold themselves responsible for their own health. The belief that becoming fat is your own responsibility links to the prevailing neo‐liberal view on health in the Netherlands; obesity is commonly framed as a matter of individual responsibility, caused by unwise food choices and a sedentary lifestyle.^34^ Another explanation could be their stage of life. Adolescence is characterized by striving for autonomy, and adults (teachers/parents) encourage adolescents to make their *own* choices and be responsible for their own behaviour.

From the data, two conflicting motives emerged. On one hand, boys need to meet Western ideals of male beauty (being muscular and neither fat nor skinny), particularly to look attractive for girls, while on the other hand boys need to meet the social norm: eating a lot, especially meat, without caring about healthy food in front of their male peers. This social norm might be stronger for boys with a low SEP than for boys with a high SEP. Although eating large portions and a lot of meat is generally seen as archetypically male,^14^ adult men with a low SEP eat more meat^35^ and less healthily^9^ than men with a high SEP. It is important that health promotion addresses the contradiction between the beauty ideal and strong social norms, since this makes it complicated for boys to eat healthily. Field and colleagues^36^ showed that almost 20% of adolescent boys have concerns about muscularity, possibly leading to psychosocial problems. A possible explanation might be that taking direct action to improve your health is still seen as a ‘women's thing,’^14^ while there is growing pressure on young men to be perfectly shaped because of the depiction of ‘perfect’ male bodies in (social) media.^16^, ^37^ Understanding the impact of the media portrayal of muscularity on young men lags behind what is known about the effect of beauty images on young women.^37^ Several recent studies call for more attention to boys in relation to satisfaction with their body.^36^, ^38^

The question is whether boys themselves find it useful to invest in health‐promotion strategies for adolescent boys. Although initial interest is low, it appears that boys do see opportunities for health promotion, particularly in direct, useful health advice that they can use to make themselves more attractive. Boys want to have a say in interventions, and autonomy matters to them. This underlines the importance of a participatory approach in interventions aimed at adolescent boys, since it gives room for autonomy and for greater empowerment.^4^, ^10^, ^24^, ^39^ Boys were open to health messages wrapped up in entertaining activities, such as cooking workshops and making vlogs. Cooking‐related activities seem especially promising.^40^ Their popularity among boys can be explained by the (social) media trend showing celebrity *male* chefs, such as Jamie Oliver and Gordon Ramsey.^14^

The challenge is to find a balance between entertainment and education in health promotion.^41^ It should be appealing to join health‐promotion activities without losing sight of educational objectives, but at the same time, education should not dominate entertainment. Future researchers might consider ‘info‐tainment’ programs, such as the creation of vlogs, for adolescent boys in a more controlled design. Possibly more important than entertainment is to collaborate with local role models and peers. Boys in disadvantaged Dutch neighbourhoods, many of whom have a migrant background, say that currently they only talk about health and health promotion with slender white (Dutch) women, and this does not work for them. For ‘Hang‐out boys’ and ‘Fitness boys’ in particular, male role models and health educators from their own neighbourhood/with the same cultural background might have more influence. Since youth centres are often visited by adolescents in Amsterdam in disadvantaged neighbourhoods, these places offer opportunities for health promotion, especially with cross‐age peers.^42^

### 4.1 Strengths and limitations

The key strength of this study is the qualitative participatory approach in which we focused on the perspectives of adolescent boys with low SEP. By using this approach, we succeeded in talking to a broad group of boys about a subject that is not of immediate interest to them. Results were used to improve health policy of the municipality of Amsterdam. Engaging this vulnerable group in public health research is important.^10^, ^17^, ^43^ However, similar to other studies,^29^, ^43^, ^44^, ^45^ it proved difficult to recruit and retain these boys, so it was important to adjust research activities to their interests and collaborate with youth workers and teachers. The use of different methods (triangulation) increased the validity of the results, but the disadvantage of using different methods and groups/sites is the relatively low number of participants per group/site. This could make generalization of the results difficult. A few girls joined in some activities, which might have influenced the results. Another limitation of the study is that the weight of the boys was not measured, but estimated by the researcher. Although this prevented stigmatization, it is possible that some boys were overweight while this was not recognized by the researcher (and vice versa). The fact that the main researcher is a woman had both advantages and disadvantages. Although a male researcher might have found it easier to connect with boys,^46^ boys often talk more openly with female researchers because male researchers might induce stereotypical masculine behaviour.^47^

This research has important implications for adolescent health promotion. Our findings offer insights into the perspectives of a seldom‐heard group and suggest better tailored interventions and directions for future research. For health promotion, it is vital to connect with who the boys are/want to be in relation to their peers. Boys mentioned that current health promotion, often provided by slender white women, does not correspond to their daily lives. The solution is to listen more to boys and tailor interventions to their specific needs and maybe also to invite more men into this field.

## ETHICAL APPROVAL

The research did not fall under the Dutch Medical Research Involving Human Subjects Act, so there was no need for official ethical approval. The ethical guidelines of the Faculty of Social and Behavioral science of the VU University Amsterdam (2016) were followed. Throughout the study, we approached informed consent as a process; consent (especially anonymity, voluntariness) was frequently discussed with the boys in addition to initial, formal written consent. We emphasized that participation was voluntary and that they had the opportunity to withdraw at any moment without giving any reason. Parents were informed by letter and asked for informed consent. We explicitly invited them to raise any questions or concerns about the project by mail, phoning/texting the researcher or via the school/welfare contact person. The research did not fall under the Dutch Medical Research Involving Human Subjects Act, so there was no need for official ethical approval. The ethical guidelines of the faculty of social sciences of the Vrije Universiteit Amsterdam were followed.

## DATA AVAILABILITY

The data that support the findings of this study are available from the corresponding author upon reasonable request.

## References

[1] Finkelstein EA , Graham W , Malhotra R . Lifetime direct medical costs of childhood obesity. Pediatrics. 2014;133(5):854‐862. 24709935 10.1542/peds.2014-0063

[2] WHO . Adolescent Obesity and Related Behaviours (2002–2014). Geneva, Switzerland: World Health Organization; 2017.

[3] Inge TH , King WC , Jenkins TM , et al. The effect of obesity in adolescence on adult health status. Pediatrics. 2013;132(6):1098‐1104. 24249816 10.1542/peds.2013-2185 PMC3838536

[4] Mcintyre P , Williams A . Adolescent Friendly Health Services An Agenda for Change Adolescent Friendly Health Services — An Agenda for Change. Geneva, Switzerland: World Health Organization; 2002.

[5] Bagherniya M , Taghipour A , Sharma M , et al. Obesity intervention programs among adolescents using social cognitive theory: a systematic literature review. Health Educ Res. 2018;33(1):26‐39. 29293954 10.1093/her/cyx079

[6] Wang Y , Lim H . The global childhood obesity epidemic and the association between socio‐economic status and childhood obesity. Int Rev Psychiatry. 2012;24(3):176‐188. 22724639 10.3109/09540261.2012.688195 PMC4561623

[7] Amsterdam Gemeente . Staat van Gezond Gewicht. Amsterdam. 2014;www.amsterdam.nl/publish/library/93/staat_van_gezond_gewicht_2014.pdf.

[8] Fahlman MM , McCaughtry N , Martin J , Shen B . Racial and socioeconomic disparities in nutrition behaviors: targeted interventions needed. J Nutr Educ Behav. 2010;42(1):10‐16. 19910257 10.1016/j.jneb.2008.11.003

[9] Darmon N , Drewnowski A . Does social class predict diet quality? Am J Clin Nutr. 2008;87(5):1107‐1117. 18469226 10.1093/ajcn/87.5.1107

[10] Kornet‐van der Aa DA , Altenburg TM , van Randeraad‐van der Zee CH , Chinapaw M . The effectiveness and promising strategies of obesity prevention and treatment programmes among adolescents from disadvantaged backgrounds: a systematic review. Obes Rev. 2017;18(5):581‐593. 28273680 10.1111/obr.12519

[11] Plachta‐Danielzik S , Pust S , Asbeck I , et al. Four‐year follow‐up of school‐based intervention on overweight children: the KOPS Study. Obesity. 2007;15(12):3159‐3169. 18198327 10.1038/oby.2007.376

[12] Bere E , Brug J , Klepp K‐I . Why do boys eat less fruit and vegetables than girls? Public Health Nutr. 2008;11(03):321‐325. 17666125 10.1017/S1368980007000729

[13] Salam RA , Das JK , Lassi ZS , Bhutta ZA . Adolescent health interventions: conclusions, evidence gaps, and research priorities. J Adolesc Health. 2016;59(4S):S88‐S92. 27664599 10.1016/j.jadohealth.2016.05.006 PMC5026678

[14] Rowlands S , Gough B . Promoting nutrition in men's health In: Rippe JM ed. Nutrition in Lifestyle Medicine. New York, NY: Humana Press; 2017:311–328.

[15] Smith JJ , Morgan PJ , Plotnikoff RC , et al. Smart‐phone obesity prevention trial for adolescent boys in low‐income communities: the ATLAS RCT. Pediatrics. 2014;134(3):e723–e731. 25157000 10.1542/peds.2014-1012

[16] Spencer RA , Rehman L , Kirk S . Understanding gender norms, nutrition, and physical activity in adolescent girls: a scoping review. Int J Behav Nutr Phys Act. 2015;12(1):6. 25616739 10.1186/s12966-015-0166-8 PMC4310022

[17] Lubans DR , Morgan PJ , Aguiar EJ , Callister R . Randomized controlled trial of the Physical Activity Leaders (PALs) program for adolescent boys from disadvantaged secondary schools. Prev Med (Baltim). 2011;52(3–4):239–246. 10.1016/j.ypmed.2011.01.009 21276812

[18] Ludvigsen A , Scott S . Real kids don't eat quiche: what food means to children. Food Cult Soc. 2009;12(4):417–436.

[19] Park S , Blanck HM , Sherry B , Brener N , O'Toole T . Factors associated with sugar‐sweetened beverage intake among United States high school students. J Nutr. 2012;142(2):306–312. 22223568 10.3945/jn.111.148536 PMC4532336

[20] Vila‐Lopez N , Kuster‐Boluda I . Adolescents food packaging perceptions. Does gender matter when weight control and health motivations are considered? Food Qual Prefer. 2016;52:179–187.

[21] Stok FM , de Ridder D , de Vet E , et al. Hungry for an intervention? Adolescents' ratings of acceptability of eating‐related intervention strategies. BMC Public Health. 2015;16(1):5. 10.1186/s12889-015-2665-6 PMC4700578 26729328

[22] Van Vliet JS , Gustafsson P , Duchén K , et al. Social inequality and age‐specific gender differences in overweight and perception of overweight among Swedish children and adolescents: a cross‐sectional study. BMC Public Health. 2015;15:628. 26156095 10.1186/s12889-015-1985-x PMC4496810

[23] McCabe MP , Ricciardelli LA . Body image dissatisfaction among males across the lifespan. J Psychosom Res. 2004;56(6):675–685. 15193964 10.1016/S0022-3999(03)00129-6

[24] Hawke LD , Relihan J , Miller J , et al. Engaging youth in research planning, design and execution: practical recommendations for researchers. Health Expect. 2018;21(6):944–949. 29858526 10.1111/hex.12795 PMC6250868

[25] MacNaughton G , Hughes P , Smith K . Young children's rights and public policy: practices and possibilities for citizenship in the early years. Child Soc. 2007;21(6):458–469.

[26] Cornwall A , Jewkes R . What is participatory research? Soc Sci Med. 1995;41(12):1667–1676. 8746866 10.1016/0277-9536(95)00127-s

[27] Baum FE . Power and glory: applying participatory action research in public health Poder y gloria: aplicación de la investigación de acción participativa en la salud pública. Gac Sanit. 2016;30(6):405–407. 27491431 10.1016/j.gaceta.2016.05.014

[28] Dedding C , Jurrius K , Moonen X , Rutjes L , Kinderen En Jongeren Actief in Wetenschappelijk Onderzoek. Houten, The Netherlands: Uitgeverij Lannoo Campus; 2013.

[29] Ellard‐Gray A , Jeffrey NK , Choubak M , Crann SE . Finding the hidden participant. Int J Qual Methods. 2015;14(5):160940691562142.

[30] Schensul SL , Schensul JJ , LeCompte MD . Essential Ethnographic Methods : Observations, Interviews, and Questionnaires. Lanham, MD: AltaMira Press; 1999.

[31] Altheide DL . Reflections: ethnographic content analysis. Qual Sociol. 1987;10(1):65–77.

[32] Berger R . Now I see it, now I don't: researcher's position and reflexivity in qualitative research. Qual Res. 2015;15(2):219–234.

[33] Whitehead M , Dahlgren G . What can be done about inequalities in health? Lancet. 1991;338(8774):1059–1063. 1681366 10.1016/0140-6736(91)91911-d

[34] Lupton D . Fat. Abingdon, UK: Routledge; 2013.

[35] Pfeiler TM , Egloff B . Examining the “Veggie” personality: results from a representative German sample. Appetite. 2018;120:246–255. 28890390 10.1016/j.appet.2017.09.005

[36] Field AE , Sonneville KR , Crosby RD , et al. Prospective associations of concerns about physique and the development of obesity, binge drinking, and drug use among adolescent boys and young adult men. JAMA Pediatr. 2014;168(1):34. 24190655 10.1001/jamapediatrics.2013.2915 PMC3947325

[37] Neumark‐Sztainer D , Eisenberg ME . Body image concerns, muscle‐enhancing behaviors, and eating disorders in males. JAMA. 2014;312(20):2156. 25423220 10.1001/jama.2014.5138

[38] Gall K , van Zutven K , Lindstrom J , et al. Obesity and emotional well‐being in adolescents: Roles of body dissatisfaction, loss of control eating, and self‐rated health. Obesity. 2016;24(4):837–842. 26880693 10.1002/oby.21428

[39] Reece LJ , Bissell P , Copeland RJ . ‘I just don't want to get bullied anymore, then I can lead a normal life ’; Insights into life as an obese adolescent and their views on obesity treatment. Health Expect. 2015;19(4):897‐907. 27403849 10.1111/hex.12385 PMC4989446

[40] Hersch D , Perdue L , Ambroz T , Boucher JL . The impact of cooking classes on food‐related preferences, attitudes, and behaviors of school‐aged children: A systematic review of the evidence, 2003–2014. Prev Chronic Dis. 2014;11:E193. 25376015 10.5888/pcd11.140267 PMC4222785

[41] Singhal A , Rogers E . Entertainment‐Education: A Communication Strategy for Social Change. London, UK: Routledge; 2012.

[42] Sato PM , Steeves EA , Carnell S , et al. A youth mentor‐led nutritional intervention in urban recreation centers: a promising strategy for childhood obesity prevention in low‐income neighborhoods. Health Educ Res. 2016;31(2):195–206. 26936480 10.1093/her/cyw011 PMC5007578

[43] Marcell AV , Klein JD , Fischer I , Allan MJ , Kokotailo PK . Male adolescent use of health care services: where are the boys? J Adolesc Health. 2002;30(1):35–43. 11755799 10.1016/s1054-139x(01)00319-6

[44] Morrison Z , Gregory D , Thibodeau Lethbridge S , Jennifer Copeland C . Ouch! Recruitment of overweight and obese adolescent boys for qualitative research. Qual Rep. 2012;17(64):1–17.

[45] Powell TW , Weeks FH , Illangasekare S , et al. Facilitators and barriers to implementing church‐based adolescent sexual health programs in Baltimore City. J Adolesc Heal. 2017;60(2):169–175. 10.1016/j.jadohealth.2016.09.017 PMC6541223 27889400

[46] Kosygina LV . The qualitative report doing gender in research: reflection on experience in field. Qual Rep. 2005;10(1):87–95.

[47] Broom A , Hand K , Tovey P . The role of gender, environment and Individual biography in shaping qualitative interview data. Int J Soc Res Methodol. 2009;12(1):51–65.

